# Comparison of the clinical features and outcome of children with hemophagocytic lymphohistiocytosis (HLH) secondary to visceral leishmaniasis and primary HLH: a single-center study

**DOI:** 10.1186/s12879-021-06408-w

**Published:** 2021-08-02

**Authors:** Hadi Mottaghipisheh, Kurosh Kalantar, Ali Amanati, Mansoureh Shokripour, Mahdi Shahriari, Omid Reza Zekavat, Soheila Zareifar, Mehran Karimi, Sezaneh Haghpanah, Mohammadreza Bordbar

**Affiliations:** 1 Hematology Research Center, Shiraz University of Medical Sciences, Shiraz, Iran; 2 Department of Immunology, School of Medicine, Shiraz University of Medical Sciences, Shiraz, Iran; 3 Professor Alborzi Clinical Microbiology Research Center, Amir Oncology Hospital, Shiraz University of Medical Sciences, Shiraz, Iran; 4 Department of Pathology, School of Medicine, Shiraz University of Medical Sciences, Shiraz, Iran; 5 Department of Pediatrics, Nemazi Hospital, Shiraz University of Medical Sciences, Shiraz, Iran

**Keywords:** Hemophagocytic lymphohistiocytosis, Visceral leishmaniosis, Prognostic factors, Survival

## Abstract

**Background:**

Hemophagocytic lymphohistiocytosis (HLH) is a syndrome of excessive inflammation. We aimed to describe the clinical and laboratory findings of HLH patients secondary to Visceral leishmaniasis (VL) and their treatment outcome during a 4-year follow-up period compared to primary HLH.

**Method:**

Forty children with primary HLH confirmed by genetic study and 20 children with HLH secondary to VL confirmed by a blood or bone marrow polymerase chain reaction from 2014 to 2018 in Shiraz, Fars province, Southern Iran, were enrolled.

**Results:**

The median age at diagnosis was 11.5 months (range 1–170), and 56.7% were male. Fever and splenomegaly were the most frequent clinical presentations. 93.3% of the subjects had an HScore > 169, which had a good correlation with HLH-2004 criteria (*r* = 0.371, *P* = 0.004). Patients with primary HLH experienced more thrombocytopenia (*P* = 0.012) and higher alanine transaminase (*P* = 0.016), while patients with VL-associated HLH had higher ferritin (*P* = 0.034) and erythrocyte sedimentation rate (*P* = 0.011). Central nervous system (CNS) involvement occurred in 38.3% of patients. The mortality rate was higher in patients with CNS disease (61% vs. 35%, *P* = 0.051). The 3-yr overall survival rate was 35.9%. (24% in primary HLH and 100% in VL-associated HLH, *P* < 0.001). In Cox regression analysis, platelet count < 100,000/ *μ* l (hazard ratio 4.472, 95% confidence interval 1.324–15.107, *P* = 0.016) correlated with increased mortality in patients with primary HLH.

**Conclusion:**

VL is a potential source of secondary HLH in regions with high endemicity. Treatment of the underlying disease in VL-associated HLH is sufficient in most cases, with no need to start etoposide-based chemotherapy.

## Introduction

Hemophagocytic lymphohistiocytosis (HLH) is a syndrome of excessive inflammation and tissue destruction due to abnormal immune activation. The absence of normal downregulation by activated macrophages and lymphocytes is possibly the cause of hyper inflammation and dysregulated immune state [1]. Infants are most commonly affected, with the highest incidence in those younger than 3 months [2].

HLH presents as a febrile illness associated with multiple organ involvement. Thus, initial signs and symptoms of HLH can mimic common infections, fever of unknown origin, hepatitis, or encephalitis [3]. Therefore, the diagnosis of HLH is primarily based on fulfilling at least 5 out of 8 criteria based on the published diagnostic criteria used in the HLH-2004 trial [4].. Similarly, homozygosity or compound heterozygosity for verified HLH-associated mutations will confirm the diagnosis [5]. Moreover, a scoring system has been developed to generate a diagnostic score referred to as “HScore,” which estimates the probability of HLH (Table 1). A score higher than 169 was shown to predict the risk of HLH with 93% sensitivity and 86% specificity [6].

**Table 1:** HScore

Parameter	Score
Known underlying immunosuppression¸ HIV positive, Immunosuppressive drugs	
No	0
Yes	18
Maximal temperature (C)	
< 38.4	0
38.4–39.4	33
> 39.4	49
Organomegaly	
No	0
Hepatomegaly or splenomegaly	23
Hepatomegaly & splenomegaly	38
Number of cytopenias (Hb ≤ 9.2 g/dL, WBC ≤ 5000/μl, PLT ≤ 110,000/μl)	
1 lineage	0
2 lineages	24
3 lineages	34
Higher ferritin level (ng/ml)	
< 2000	0
2000–6000	35
> 6000	50
Higher triglyceride level (mg/dL)	
< 133.5	0
133.5–356	44
> 356	64
Lower fibrinogen level (g/L)	
> 250	0
≤ 250	30
Higher SGOT/AST level (IU/L)	
< 30	0
≥ 30	19
Hemophagocytosis features on bone marrow aspirate	
No	0
Yes	35
Total score	

*HIV* human immunodeficiency virus, *Hb* hemoglobin, *WBC* white blood cell, *PLT* platelet, *SGOT* serum glutamic-oxaloacetic transaminase, *AST* aspartate aminotransferase

On the other hand, secondary (sporadic, acquired) HLH is generally used to describe patients without a known familial mutation who typically have a clear trigger for developing HLH. It usually follows an infectious disease caused by organisms, including Epstein-Barr virus (EBV), cytomegalovirus (CMV), hepatitis viruses, varicella infection, leishmania, *Mycobacterium tuberculosis*, among many others. Moreover, autoimmune and rheumatologic disorders, malignancies, and metabolic disorders may trigger the cytokine storm [7].

A few small case series of primary or secondary HLH have been reported in Iran [8–10]. Additionally, despite a high prevalence of Visceral leishmaniasis (VL) in Southern Iran [11], there are few reports of VL-associated HLH in our region [12, 13]. Therefore, in this prospective study, we aimed to describe the clinical and laboratory findings and the outcome of children diagnosed with VL-associated HLH during a 4-year follow-up period compared to patients with primary HLH.

## Methods and materials

### Study population and data acquisition

This prospective observational study was conducted in a tertiary oncology referral center in Shiraz, Fars province in Southern Iran, 2014–2018. During the study period, patients with a confirmed diagnosis of HLH were included. HLH was diagnosed based on the diagnostic criteria proposed by the Histiocyte Society in 2004 [14].

Patients with a positive family history or with disease-causing mutations in the genes encoding perforin (FHL2), Munc 13–4 (FHL3), Syntaxin 11 (FHL4), Munc 18–2 (FHL5), Lyst (Chediak-Higashi syndrome), and Rab 27A (Griscelli syndrome type 2), signaling lymphocyte activation molecule associated protein (XLP1) and X-linked inhibitor of apoptosis (XLP2) were defined as primary HLH. Patients with a confirmed VL diagnosis with a blood or bone marrow polymerase chain reaction (PCR) or indirect fluorescent antibody (IFA) test who fulfilled HLH-2004 criteria were defined as VL-associated HLH. In addition, information on demographic characteristics, clinical, laboratory, and radiological findings at presentation, treatment response, and survival outcomes were collected. HScore was calculated in each patient to estimate the probability of HLH in our patients and compare it with HLH-2004 diagnostic criteria. Participants in this study were assented to come in the survey according to the local Ethics Committee of the Shiraz University of Medical Sciences (IR.sums.med.rec. 1396.s195). A written informed consent was signed by the parents or the legal guardians of the participants.

### Initial diagnostic workup

Based on our HLH-management protocol, the initial diagnostic workup included serological tests for EBV, CMV, human immunodeficiency virus, Brucella, Salmonella, and VL. In any suspected case of viral-associated HLH or leishmania-associated HLH, an additional molecular test with real-time polymerase chain reaction (RT-PCR) was requested to quantify viral or parasite load. RT-PCR provides valuable information about baseline virologic or parasitic status and is very useful for monitoring and response to treatment. The molecular diagnosis of disease was assessed with whole-exome sequencing with the next-generation sequencing (NGS) method in most study patients. The mutations found by NGS were confirmed by Sanger method.

### Soluble CD25 (sCD25) measurement

sCD25 was measured using a commercial ELISA kit (Human sCD25 ELISA Ready-SET-Go, e-bioscience, USA) (sensitivity: 4 pg/ml) based on the manufacturer’s instructions. We determined the levels of sCD25 within the sample by setting up a standard curve of known target protein concentrations provided in the kit. Normal ranges for sCD25 in this study were 1.9–13.1 pg/ml.

### CNS involvement

CNS involvement was defined if the patients had neurologic signs and symptoms including seizure, altered level of consciousness, or focal neurologic signs in addition to one of these two conditions: 1. pleocytosis and/or proteinosis in cerebrospinal fluid or 2. radiologic abnormalities on magnetic resonance imaging (MRI) such as high signal intensity lesions, hemorrhage, atrophy, and leptomeningeal enhancement.

### Treatment protocol

All of our study patients with primary HLH were treated with HLA-94-based immune-chemotherapy. Briefly, the induction regimen lasted 8 weeks, including etoposide 150 mg/m^2^/day twice a week for 2 weeks and then once weekly for the next 6 weeks. Besides, dexamethasone 10 mg/m^2^/day for 2 weeks was started. The dose was halved every 2 weeks and tapered and discontinued on the last week of induction therapy. Patients with evidence of CNS disease were additionally treated with intrathecal methotrexate if the symptoms did not improve with systemic treatment. The continuation therapy included etoposide 150 mg/m^2^/day every other week with pulses of dexamethasone 10 mg/m^2^/day for 3 days and oral cyclosporine 6 mg/kg/day aiming at blood trough level around 200 mcg/L. The treatment continued for up to 40 weeks unless hematopoietic stem cell transplantation from a matched donor was available. Most of the patients with leishmaniasis-associated HLH (94.7%) were only treated with liposomal amphotericin B (total cumulative dose: 20 mg/kg divided into 4 or 5 doses administered in 4 or 5 days, respectively with no need to start HLH-specific therapy [15].. A minority were treated with dexamethasone (0.15 mg/kg/dose three times a day) for 3–5 days to control the cytokine storm.

### Statistical analysis

Data were analyzed by the Statistical Package for the Social Sciences (SPSS Inc., Chicago, Illinois, USA, version 23). Descriptive data were presented as median, interquartile range (IQR), and percentages. The comparison of qualitative variables among different groups was made by the Chi-square test or the Fisher exact test. Quantitative variables with non-normal distribution were compared with the non-parametric Mann-Whitney U test between two groups. The Pearson correlation tested the correlation between the 2004-HLH diagnosis criteria and HScore. Univariate analysis was used to test possible covariates associated with outcome measures, including CNS involvement, relapse, and death. Those variables with a *P* value less than 0.2 were entered in multivariable analysis. Binary logistic regression was used to determine independent variables associated with CNS involvement and relapse. The survival curve was illustrated by the Kaplan-Mayer method. The Log-rank test evaluated a comparison of survival curves between two groups of patients. The Cox regression model was used to determine the independent variables associated with the outcome. The associations were reported as hazard ratio (HR) and 95% confidence interval (CI). A *P*-value of less than 0.05 was considered statistically significant.

## Results

### Demographic data

Sixty patients with a confirmed diagnosis of HLH were enrolled. The clinical features and laboratory findings of patients at their initial presentation are shown in Table 2. In total, 34 patients were male with male/ female ratio = 1.30. The median age at diagnosis was 11.5 months (IQR 4–35.7 months), and 42 patients (70%) were younger than 24 months. Twenty patients (33.3%) had VL-associated HLH.

**Table 2:** Clinical features and laboratory data of patients with primary and visceral leishmaniosis-associated hemophagocytic lymphohistiocytosis

Variables		HLH classification		*P*-Value
	All patients (*n* = 60) n (%)	Primary HLH (*n* = 40) n (%)	VL-associated HLH (*n* = 20) n (%)	
Age				
<24 months	42 (70)	27 (67.5)	15 (75)	0.550
≥ 24 months	18 (30)	13 (32.5)	5 (25)	
Sex				
Male	34 (56.7)	24 (60)	10 (50)	0.461
Female	26 (43.3)	16 (40)	10 (50)	
Fever				
<30 days	42 (70)	27 (67.5)	15 (75)	>0.999
≥ 30 days	18 (30)	13 (32.5)	5 (25)	
Hepatomegaly	36 (60)	24 (60)	12 (60)	>0.999
Splenomegaly	58 (96.7)	39 (97.5)	19 (95)	>0.999
Skin lesions	5 (8.3)	5 (12.5)	0 (0)	0.159
Bleeding tendency	5 (8.3)	5 (12.5)	0 (0)	0.159
CNS Involvement	23 (38.3)	18 (45)	5 (25)	0.133
Abnormal brain MRI	9 (15)	8 (20)	1 (5)	0.249
Bone marrow hemophagocytosis	6 (10)	6 (15)	0 (0)	0.165
Relapse	6 (10)	6 (15)	0 (0)	0.165
Death	26 (43.3)	26 (65)	0 (0)	< 0.001^*^
H-score				
≥ 169	56 (93.3)	37 (92.5)	19 (95)	> 0.999
< 169	4 (6.7)	3 (7.5)	1 (5)	
ANC <1000/μl	51 (85)	34 (85)	17 (85)	>0.999
Hemoglobin <9 g/dL	42 (70)	28 (70)	14 (70)	>0.999
Platelet <100000/μl	40 (66.7)	31 (77.5)	9 (45)	0.012^*^
Bicytopenia	48 (80)	34 (85)	14 (70)	0.304
Triglyceride >265 mg/dL	41 (68.3)	28 (70)	13 (65)	0.695
Fibrinogen <150 mg/dL	32 (53.3)	20 (50)	12 (60)	0.464
Ferritin				
500-1000 ng/ml	3 (5)	3 (7.5)	0 (0.0)	0.147
1000-10,000 ng/ml	41 (68.3)	29 (72.5)	12 (60)	
>10000 ng/ml	16 (26.7)	8 (20)	8 (40)	
Ferritin, (ng/ml) median (IQR)	4825 (2000.0-10,324.5)	3618 (1990.2-9479.2)	7122 (3226.2-33,112.2)	0.034^*^
D-dimer > 250 ng/ml	59 (98.3)	39 (97.5)	20 (100)	>0.999
PT Prolonged (age adjusted)	24 (40)	15 (37.5)	9 (45)	0.576
aPTT Prolonged (age adjusted)	25 (41.7)	18 (45)	7 (35)	0.459
AST >2 × ULN (IU/L)	43 (71.7)	30 (75)	13 (65)	0.418
ALT >2 × ULN (IU/L)	25 (41.7)	21 (52.5)	4 (20)	0.016^*^
ESR > 20 mm/hr	31 (51.7)	16 (40)	15 (75)	0.011^*^
CRP ≥ 6 mg/L	53 (88.3)	33 (82.5)	20 (100)	0.084
^**^sCD25 ≥ 13.1 pg/mL	14 (70)	9 (64.3)	5 (83.3)	0.613

*VL* visceral leishmaniosis, *HLH* hemophagocytic lymphohistiocytosis, *ULN* upper limit of normal, *ANC* absolute neutrophil count, *PT* prothrombin time, *aPTT* activated partial thromboplastin time, *AST* aspartate aminotransferase, *ALT* alanine aminotransferase, *LDH* lactic dehydrogenase, *ESR* Erythrocyte sedimentation rate, *CRP* C-reactive protein, *CNS* central nervous system, *sCD25* soluble CD25

^*^Statistically significant

^**^sCD25 was measured in 20 patients

### Clinical and laboratory data

Fever and splenomegaly comprised the most frequent initial clinical findings, with 100 and 96.7% prevalence, respectively. Similarly, high serum ferritin (> 500 ng/ml) and high serum lactic dehydrogenase (LDH) (> 450 U/dL) were found exclusively in all patients. The study population’s median serum ferritin was 4825 ng/ml (range 912–72,000). Extreme hyperferritinemia (> 10,000 ng/ml) was detected in 16 (26.7%) of individuals. sCD25 was measured in one-third of the study population. It was increased in 14 (70%) out of 20 individuals.

The clinical and laboratory characteristics of patients with primary and VL-associated secondary HLH were compared in Table 2. Patients with primary HLH had a more frequently low platelet count of less than 100,000/μl (*P* = 0.012) and increased alanine transaminase (ALT) (*P* = 0.016) compared to secondary HLH. Moreover, the risk of death was significantly higher in primary HLH (*P* < 0.001). On the other hand, patients with VL-associated HLH had higher serum ferritin (*P* = 0.034). Moreover, they included a higher proportion of patients with high ESR (> 20 mm/hr) than primary HLH (*P* = 0.011). None of the patients in this group showed skin eruptions, bleeding tendency, or bone marrow hemophagocytosis. Nobody experienced disease relapse, and all of them survived.

### HScore

Fifty-six (93.3%) patients achieved a score ≥ of 169, with no difference in the two groups of primary and secondary HLH (*P* > 0.99). HScore significantly correlated with HLH-2004 criteria (*r* = 0.371, *P* = 0.004).

### CNS involvement

CNS involvement occurred in 23 (38.3%) patients. The proportion of patients with CNS involvement was not significantly different between primary and secondary HLH groups (*P* = 0.133). In univariate analysis, age < 2 years (*P* = 0.024), bone marrow hemophagocytosis (*P* = 0.027), and bicytopenia (*P* = 0.02) were associated with CNS involvement (Table 3). In subgroup analysis, age < 2 years remained a risk factor in primary HLH (*P* = 0.016). However, no risk factor was found in patients with VL-associated HLH. Binary logistic regression revealed that only age < 2 years (Odds ratio (OR) 6.446, 95% CI 1.538–27.012, *P* = 0.011) increased the odds of CNS involvement. On the other hand, patients with bicytopenia had less chance of neurologic disease (OR 0.073, 95% CI 0.008–0.838, *P* = 0.018). Patients with CNS disease had a worse outcome than those without CNS involvement (death rate 61% vs. 35%) with marginal statistical significance (*P* = 0.051).

**Table 3:** Univariate analysis of covariates associated with central nervous system involvement

CNS involvement	Total		*P*-Value	Primary HLH		*P*-Value	VL-associated HLH		*P*-Value
Variables	Yes (*n* = 23) n (%)	No (*n* = 37) n (%)		Yes (*n* = 18)	No (*n* = 22)		Yes (*n* = 5)	No (*n* = 15)	
Age									
<24 months	20 (87)	22 (59.5)	0.024^*^	16 (88.9)	11 (50)	0.016^*^	4 (80)	11 (73.3)	> 0.999
≥ 24 months	3 (13)	15 (40.5)		2 (11.1)	11 (50)		1 (20)	4 (26.7)	
Sex									
Male	16 (69.6)	18 (48.6)	0.112	14 (77.8)	10 (45.5)	0.054	2 (40)	8 (53.3)	> 0.999
Female	7 (30.4)	19 (51.4)		4 (22.2)	12 (54.5)		3 (60)	7 (46.7)	
Platelet <100000/μl	18 (78.3)	22 (59.5)	0.133	15 (83.3)	16 (72.7)	0.476	3 (60)	6 (40)	0.617
Bicytopenia	22 (95.7)	26 (70.3)	0.020^*^	17 (94.4)	17 (77.3)	0.197	5 (100)	9 (60)	0.260
Fibrinogen <150 mg/dL	9 (39.1)	23 (62.2)	0.082	7 (38.9)	13 (59.1)	0.341	2 (40)	10 (66.7)	0.347
ALT>2 × ULN (IU/L)	12 (52.2)	13 (35.1)	0.193	12 (66.7)	9 (40.9)	0.125	0 (0)	4 (26.7)	0.530
BM. hemophagocytosis	5 (21.7)	1 (2.7)	0.027^*^	5 (27.8)	1 (4.5)	0.073	0 (0)	0 (0)	–
Bleeding tendency	4 (17.4)	1 (2.7)	0.066	4 (22.2)	1 (4.5)	0.155	0 (0)	0 (0)	–

*ULN* upper limit of normal, *ALT* alanine aminotransferase, *BM* bone marrow, *CNS* central nervous system

^*^statistically significant

### Treatment and outcomes

During the study period, 33 patients (55%) showed a treatment response. Twenty-nine patients (87%) finished their treatment successfully. They were all alive and off-treatment at the end of the study. Treatment was ongoing in 4 more patients with an excellent initial response to treatment. Twenty-six (43.3%) patients died during treatment that all of them had primary HLH.

**Fig. 1:**
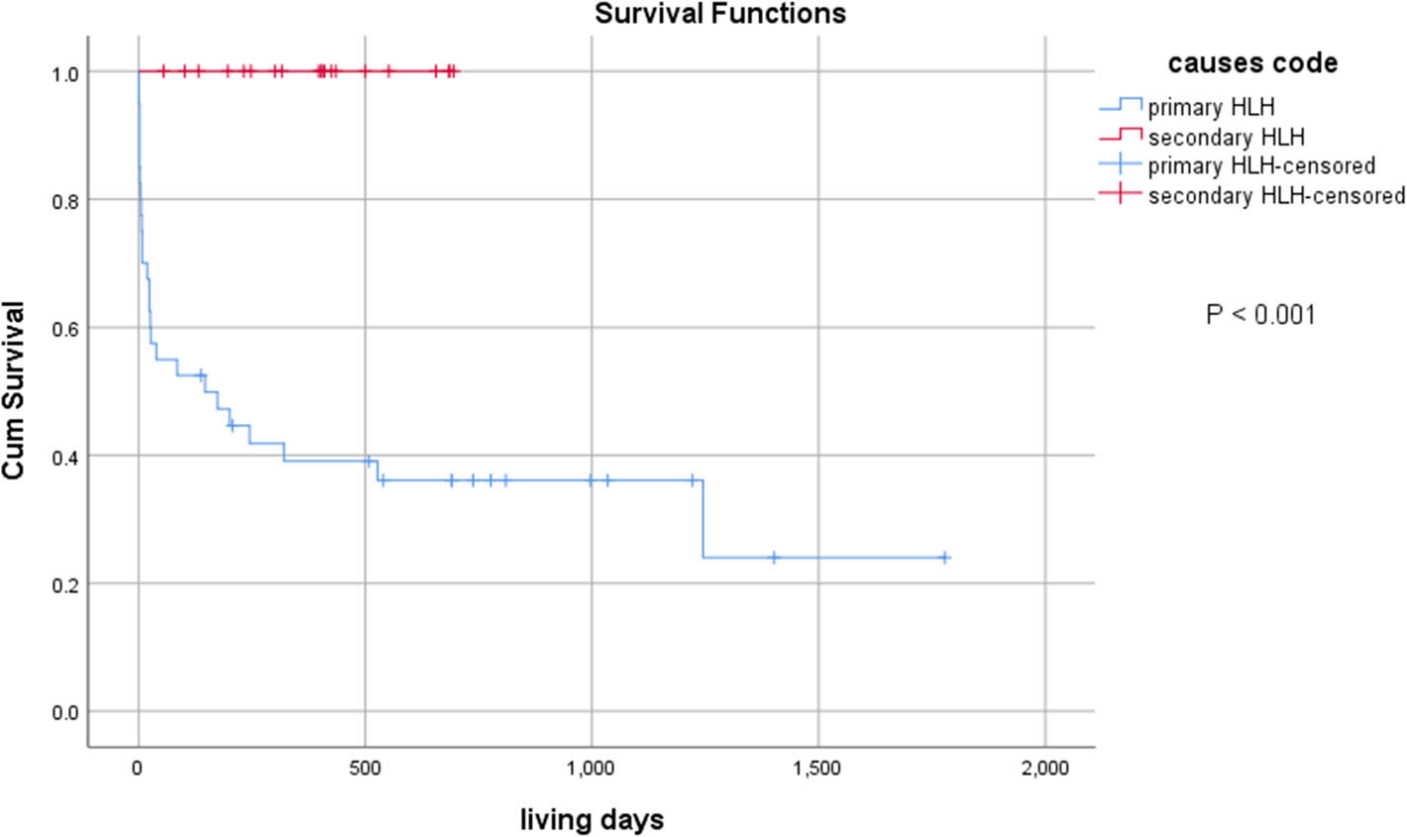
Kaplan-Meier survival curve of patients with hemophagocytic lymphohistiocytosis

Fig. 1 shows the Kaplan-Meier survival curve of the study population during the 40 months follow-up period. The patients experienced a 3-year overall survival (OS) rate of 35.9%, which was significantly lower in the primary HLH than the secondary HLH group (24% vs. 100%; *P* < 0.001).

Table 4 demonstrates a univariate analysis of possible covariates associated with the outcome in patients with HLH, assessed by the log-rank test. Among the tested variables, platelet count < 100,000 /μl (*P* = 0.001), total bilirubin > 1 mg/dL (*P* = 0.008), and erythrocyte sedimentation rate (ESR) ≤ 20 mm/hr. (*P* = 0.02) were associated with mortality. In Cox regression analysis, platelet count < 100,000/ *μ* l (HR 4.472, 95% CI 1.324–15.107, *P* = 0.016) remained an independent risk factor of mortality.

**Table 4:** Univariate analysis of covariates associated with mortality in patients with primary hemophagocytic lymphohistiocytosis

Variables	Mean survival in days (95% confidence interval)	*P*-value
Splenomegaly		
Yes	935.9 (678.5-1193.4)	0.069
No	37 (1.72-72.3)	
BM. hemophagocytosis		
Yes	146.0 (24.9-267.1)	0.059
No	977.2 (710.2-1244.2)	
CNS involvement		
Yes	655.6 (304.0-1007.0)	0.092
No	849.3 (657.7-1040.9)	
Bleeding tendency		
Yes	195.8 (24.5-367.1)	0.059
No	960.1 (695.9-1224.2)	
Bicytopenia		
Yes	801.7 (540.0-1063.4)	0.052
No	845.2 (652.5-1037.8)	
Platelet count		
<100,000/μL	711.2 (443.8-978.7)	0.001*
≥ 100,000/μL	1195.2 (1001.1-1389.3)	
Hemoglobin		
< 9 g/dL	844.0 (562.9-1125.2)	0.188
≥ 9 g/dL	808.1 (547.3-1068.9)	
Total bilirubin		
> 1 mg/dL	571.2 (275.5-867.0)	0.008*
≤ 1 mg/dL	1004.3 (781.7-1226.8)	
ESR		
> 20 mm/hr	1271.7 (934.8-1608.6)	0.002*
≤ 20 mm/hr	459.4 (298.7-620.1)	
Fibrinogen		
< 150 mg/dL	1001.8 (660.1- 1343.5)	0.183
≥ 150 mg/dL	658.7 (405.4-912.1)	

*ULN* upper limit of normal, *ALT* alanine aminotransferase, *ESR* Erythrocyte sedimentation rate, *CNS* central nervous system, *BM* bone marrow

†The data were analyzed by the Log-rank test; only variables with *P* < 0.2 are shown

*Statistically significant

During the 40 months follow-up period, six patients (10%) experienced disease relapse. All of them occurred in boys with primary HLH. In multivariable analysis, no independent risk factor was found.

## Discussion

The current study represents the first study on HLH patients in a large referral oncology center in Southern Iran. It is also one of the most extensive case series of secondary HLH associated with VL. It was reported that VL is associated with about 2% of all cases of HLH [16]. However, we encountered a much higher prevalence due to VL’s high endemicity in Southern Iran, where this study was undertaken. It is good to know that Fars province is one of the central endemic regions of visceral leishmaniasis in southern Iran, with an estimated incidence rate of 0.01/10,000 in 2015 [11]. A recent meta-analysis reported a pooled prevalence of 2.1% (95% CI 1.4–2.8%) of VL in Southern Iran [17]. The first report of a benign hemophagocytic syndrome associated with VL in our region was published by Kumar et al. in 1994 [12]. Mokhtari and Kumar reported the first case series of VL-associated HLH among 13 patients and described their bone marrow characteristics. Hypercellular marrow with erythroid hyperplasia, megaloblastic changes, foamy macrophages, activated macrophages with cytoplasmic vacuoles and elongated cytoplasmic process, intra- and extracellular amastigotes, cytoplasmic fragments (blue bodies), plasma cells with inclusions and hemophagocytic cells, in addition to Leishman bodies (amastigotes) were the most common microscopic findings of the studied patients. The most common hematological abnormalities were anemia (88.8%), thrombocytopenia (50%,) and pancytopenia (55.5%) [13].

The clinical sign and symptoms and laboratory parameters in HLH associated with VL were very similar to primary HLH. Fever and splenomegaly were the most consistent presenting signs and symptoms detected in nearly all patients. Serum ferritin at diagnosis was higher than 900 ng/ml in all patients, and about 30% of them had extreme hyperferritinemia (> 10,000 ng/ml). However, it did not correlate with disease severity or adverse outcomes such as death or relapse. Allen et al. reported that ferritin level over 10,000 ng/ml was 90% sensitive and 96% specific for HLH [18].

On the other hand, a recent review concluded that HLH is a rare cause of extreme hyperferritinemia, and more common causes such as liver disease and malignancies should be ruled out first [19]. Since timely diagnosis and treatment are directly related to disease outcome, high serum ferritin should raise suspicion to HLH diagnosis if other criteria are met, especially in critically ill patients.

Patients with VL-associated HLH experienced higher serum ferritin and ESR. Though the underlying infection may be a simple explanation, enhanced ferroportin-mediated iron efflux due to overexpression of growth differentiation factor 15 may play a role [20]. On the other hand, primary HLH was associated with more severe thrombocytopenia, higher ALT, and worse outcome than secondary HLH. Koh et al. reported that splenomegaly, bicytopenia, Hb < 9 g/dL, and platelets < 100,000/μl were more frequently encountered in patients with primary HLH. They also reported a lower 5-yr OS in primary HLH compared to secondary HLH [21]. Some of the mentioned features, such as bicytopenia and thrombocytopenia, can be regarded as markers of disease severity, directly linked to poor survival. We came across a similar result in which thrombocytopenia served as an independent risk factor of death, especially in patients with primary HLH.

Nearly 40% of our patients had evidence of CNS involvement. The rate of CNS involvement in HLH varies in different studies ranging from 18 to 73% [22]. Some reported previously that patients with CNS disease had lower ferritin, AST, and ALT than those without CNS involvement [23]. We did not find such an association in our patients except that children younger than 2 years had more than a 6-fold higher chance of CNS disease than older patients. Moreover, bicytopenia decreased the probability of CNS involvement by 93%.

Many reports are indicating that CNS involvement might be associated with worse outcomes (19). Although the overall mortality rate was higher in those with CNS events, this association was not shown in multivariable analysis. It is generally agreed that CNS involvement poses a higher risk of relapse unless hematopoietic stem cell transplantation (HSCT) is undergone early after starting systemic therapy [22].

HScore had a good correlation with HLH-2004 criteria for the diagnosis of HLH in our study. It was reported that HScore carries a diagnostic sensitivity of 90% and specificity of 79% for HLH [6]. A cutoff of 169 had 93% sensitivity and 86% specificity for HLH [24]. Similarly, more than 90% of our confirmed cases of HLH had HScore ≥169. Given the unavailability of sCD25 and natural killer (NK) cell activity in some centers, HScore can be utilized to make early diagnosis and treatment in case of incomplete criteria.

The treatment response was 55% in our study cohort with a 3-yr OS rate of 36%. It was much lower in patients with primary HLH than secondary HLH (24% vs. 100%). All patients with VL-associated HLH were successfully treated with liposomal amphotericin. Nobody needed to be treated with etoposide or cyclosporine. Dexamethasone was initially started in a few patients with severe disease but was tapered and discontinued after a few weeks. The outcome was excellent, with no report of death or HLH relapse during the 40 months follow-up. Targeted treatment of the etiologic pathogen usually is the only treatment strategy recommended in secondary infection-associated HLH such as brucella and leishmania. Specific antimicrobial therapy usually is associated with complete recovery in most cases [25–31].

High-dose intravenous gamma-globulin (IVIG) has been used successfully in viral-associated hemophagocytic syndrome [32]; however, none of our cases were treated with IVIG.

Platelet count less than 100,000/μl was independently associated with more than four times the increased risk of death in our patients with primary HLH. The outcome of patients with HLH and associated prognostic factors differ among different studies. A recent study in Japan reported a much better outcome in their patients with a 3-yr OS rate of 74% [33]. Koh et al. reported a 5-yr OS rate of 68% in their study cohort, which was worse (38%) in primary HLH patients. They concluded that lower age at diagnosis, severe transaminasemia, and coagulation abnormality were independent risk factors of survival [21]. In other studies, decreased serum albumin level, LDH ≥ 3707 U/L, IL-10 ≥ 456 pg/ml, neutrophil count < 500/μl, and total bilirubin > 2× normal upper limit was associated with a higher mortality rate [34–36].

Our study faced some limitations. Firstly, some specific laboratory tests, such as NK cell function, were unavailable in our center. Besides, sCD25 was measured in a portion of our patients since it was available in our center in 2017. These tests’ unavailability might lead to a delayed diagnosis and poor outcome, evident as a lower 3-yr OS rate in our study cohort than many other reports.

## Conclusion

VL is a potential source of secondary HLH in regions with high endemicity. The main clinical findings and complications such as CNS involvement are the same as primary HLH. It is associated with higher serum ferritin and ESR but a lower incidence of thrombocytopenia and transaminitis than primary HLH. Treatment of the underlying disease in VL-associated HLH is sufficient in most cases, with no need to start etoposide-based chemotherapy. The prognosis is excellent, with no reported death or recurrence of disease in our case series.

## Data Availability

The data presented in this study are available on request from the corresponding author.

## Abbreviations

HLH: Hemophagocytic lymphohistiocytosis
VL: Visceral leishmaniasis
CNS: Central nervous system
EBV: Epstein-Barr virus
CMV: Cytomegalovirus
RT-PCR: Real-time polymerase chain reaction
sCD25: Soluble CD25
MRI: Magnetic resonance imaging
SPSS: Statistical Package for the Social Sciences
IQR: Interquartile range
CI: Confidence interval
HR: Hazard ratio
ALT: Alanine transaminase
AST: Aspartate transaminase
OR: Odds ratio
NK cell: Natural killer cell
OS: Overall survival
LDH: Lactate dehydrogenase
IL-10: Interleukin 10
HSCT: Hematopoietic stem cell transplantation
ESR: Erythrocyte sedimentation rate
ULN: Upper limit of normal
BM: Bone marrow
HIV: Human immunodeficiency virus
aPTT: Activated partial thromboplastin time
PT: Prothrombin time
SGOT: Serum glutamic-oxaloacetic transaminase
HB: Hemoglobin
WBC: White blood cell
PLT: Platelet
ANC: Absolute neutrophil count
CRP: C-reactive protein
